# miR-196b-Oct1/2 axis regulates *DNMT3A*-mutant AML pathogenesis

**DOI:** 10.1038/s41375-024-02456-8

**Published:** 2024-11-23

**Authors:** Michael E. Lawler, Melanie L. Goetz, Jennifer S. Romer-Seibert, Holly A. Gamlen, Edwina McGlinn, Sara E. Meyer

**Affiliations:** 1 Department of Pharmacology, Physiology, and Cancer Biology, Sidney Kimmel Comprehensive Cancer Center, Thomas Jefferson University, Philadelphia, PA USA; 2 EMBL Australia, Australian Regenerative Medicine Institute, Monash University, Clayton, VIC Australia; 3 Department of Medical Oncology, Sidney Kimmel Comprehensive Cancer Center, Thomas Jefferson University, Philadelphia, PA USA

**Keywords:** Acute myeloid leukaemia, Cancer models

## To the Editor

Somatic mutations in the de novo DNA methyltransferase *DNMT3A* occur in approximately one-third of cytogenetically normal acute myeloid leukemia (AML) [1]. *DNMT3A* mutations result in global DNA hypomethylation, consistent with DNMT3A loss-of-function, and abnormal self-renewal of hematopoietic stem cells [2]. While DNA hypomethylation is reversible and required to maintain the leukemogenic potential of *DNMT3A*-mutant AML cells [3], the loss of *Dnmt3a* alone is not sufficient for leukemic transformation of normal hematopoietic stem and progenitor cells in mice [4]. In AML, mutations in epigenetic modifiers are almost exclusively found with additional genetic lesions. Constitutive activating mutations in the receptor tyrosine kinase *FLT3*, commonly internal tandem duplications (ITD) that result in ligand-independent receptor activation, frequently co-occur with *DNMT3A* mutations [1, 5]. Consistent with this, we previously reported that mice with combined somatic loss of *Dnmt3a* and germline knock-in of *Flt3*^ITD^ (*Dnmt3a*^+/-^*Flt3*^*ITD*^) develop fully penetrant, transplantable, lethal AML [3]. However, identification of therapeutically targetable pathways in *DNMT3A*-mutant AML remain a major challenge as differences in DNA methylation have only modest concordance with  protein-coding gene expression.

Deregulation of microRNA (miRNA) is a common pathogenic mechanism in human malignancies, including AML [6]. We previously reported that miR-196b is hypomethylated and overexpressed in human and murine *DNMT3A*-mutant AML and is associated with poor survival [7]. We discovered that miR-196b mediated repression of Toll-like-receptor (TLR) signaling is important for *DNMT3A*-mutant human AML and *Dnmt3a*^+/-^*Flt3*^*ITD*^ murine AML by dampening the ability of AML cells to mature in response to TLR stimuli [7]. However, whether miR-196b is required for leukemia initiation and development by somatic *DNMT3A* loss-of-function mutations is not known.

**Fig. 1:**
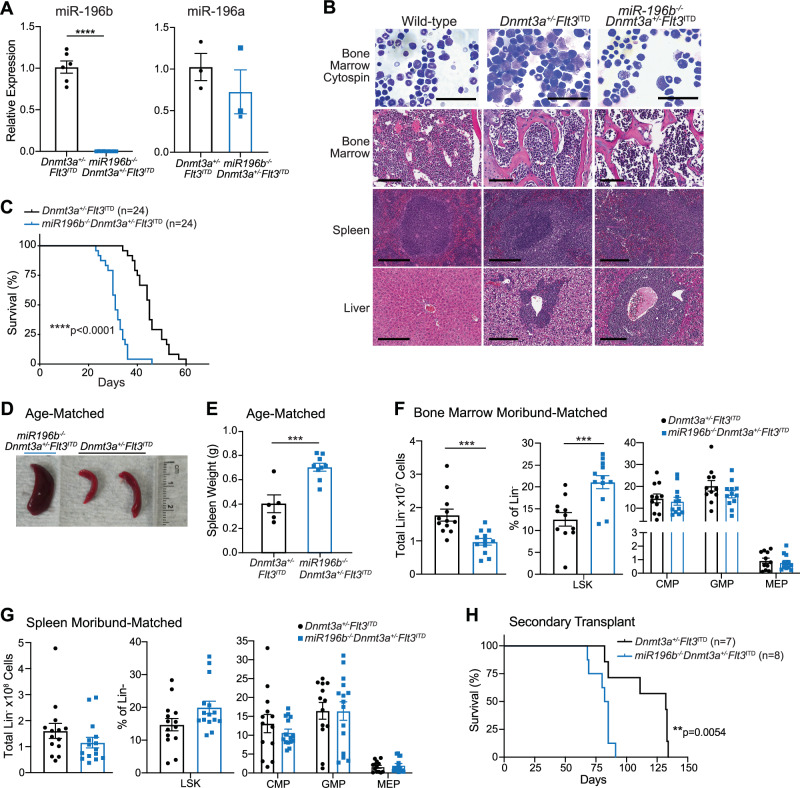
miR-196b is important for leukemia development in *Dnmt3a*/*Flt3*-mutant mice. **A** RT-qPCR analysis of average miR-196b and miR-196a expression ± SEM in *miR196b*^*–/–*^*Dnmt3a*^*+/–*^*Flt3*^*ITD*^ AML relative to *Dnmt3a*^*+/–*^*Flt3*^*ITD*^ AML. Significance determined by unpaired *t* test. **B** Representative images of Wright-Giemsa-stained bone marrow cytospins and H&E-stained bone marrow, spleen, and liver samples from moribund *miR196b*^*–/–*^*Dnmt3a*^*+/–*^*Flt3*^*ITD*^ and age-matched *Dnmt3a*^*+/-*^*Flt3*^*ITD*^ at 26 days and normal wild-type (WT) at 24 days. Scale bars for cytospin, bone marrow, spleen, and liver represent 50 mm, 80 mm, 300 mm, and 200 mm, respectively. Bone marrow shows numerous myeloblasts and immature myeloid progenitors in *miR196b*^*-/-*^*Dnmt3a*^*+/–*^*Flt3*^*ITD*^ and *Dnmt3a*^*+/–*^*Flt3*^*ITD*^ samples. Liver and spleen of *miR196b*^*–/–*^*Dnmt3a*^*+/–*^*Flt3*^*ITD*^ and *Dnmt3a*^*+/–*^*Flt3*^*ITD*^ show disruption of normal architecture due to infiltration of myeloid blasts and progenitor cells. **C** Kaplan-Meier survival analysis of *Dnmt3a*^*+/*^–*Flt3*^*ITD*^ mice (*n* = 24) compared to *miR196b*^*–/–*^*Dnmt3a*^*+/–*^*Flt3*^*ITD*^ mice (*n* = 24). Median ages of moribund *miR196b*^*–/–*^*Dnmt3a*^*+/–*^*Flt3*^*ITD*^ and *Dnmt3a*^*+/–*^*Flt3*^*ITD*^ mice were 33 and 41 days, respectively. Significance determined by the log-rank (Mantel-Cox) test. **D** Representative image of spleens harvested from a moribund *miR196b*^*–/–*^*Dnmt3a*^*+/–*^*Flt3*^*ITD*^ AML mouse and two age-matched *Dnmt3a*^*+/-*^*Flt3*^*ITD*^ AML mice. **E** Average spleen weights of moribund *miR196b*^*–/–*^*Dnmt3a*^*+/–*^*Flt3*^*ITD*^ mice (*n* = 9) compared to age-matched *Dnmt3a*^*+/*^–*Flt3*^*ITD*^ mice (*n* = 5). Median ages of *miR196b*^*–/–*^*Dnmt3a*^*+/–*^*Flt3*^*ITD*^ and *Dnmt3a*^*+/–*^*Flt3*^*ITD*^ mice were 30 and 29 days, respectively. Significance determined by unpaired *t* test. Flow cytometric analyses of average total number ±SEM of lineage^-^ (Lin-) cells and average frequencies ± SEM of Lin^-^Sca1^+^c-Kit^+^ (LSK), common myeloid (CMP), granulocyte-monocyte (GMP), and megakaryocyte-erythroid (MEP) progenitor cells isolated from bone marrow (**F**) and spleens (**G**) of moribund *Dnmt3a*^*+/-*^*Flt3*^*ITD*^ (bone marrow *n* = 11, spleen *n* = 14) and *miR196b*^*-/-*^*Dnmt3a*^*+/-*^*Flt3*^*ITD*^ (bone marrow *n* = 12, spleen *n* = 15) AML mice. Significant differences were evaluated by unpaired *t* tests for Lin- and LSK and by two-way ANOVA Šídák’s multiple comparisons test for CMP, GMP, MEP populations. **H** Kaplan-Meier survival analysis of secondary recipient mice transplanted with c-Kit^+^ *Dnmt3a*^*+/–*^*Flt3*^*ITD*^ (*n* = 7) or *miR196b*^–*/–*^*Dnmt3a*^*+/–*^*Flt3*^*ITD*^ (*n* = 8) AML. Significance determined by the log-rank (Mantel-Cox) test.

To investigate the role of miR-196b in leukemia initiation by mutant *Dnmt3a*, we generated *Dnmt3a*^+/–^*Flt3*^*ITD*^ mice with germline deletion of miR-196b [8] (*miR196b*^*–/–*^*Dnmt3a*^+/–^*Flt3*^*ITD*^). The deletion is specific to miR-196b and does not significantly alter miR-196a expression (Fig. 1A). Of note, miR-196b deletion alone does not perturb normal steady state hematopoiesis in mice (Supplementary Fig. 1A–H). As previously reported, *Dnmt3a*^+/-^*Flt3*^*ITD*^ mice develop AML [3] as evidenced by increased immature forms in the bone marrow and myeloblastic infiltration in liver and spleen as compared to wild-type control mice (Fig. 1B). *miR196b*^*-/-*^*Dnmt3a*^+/-^*Flt3*^*ITD*^ mice also develop AML with significantly shorter latency compared to *Dnmt3a*^+/–^*Flt3*^*ITD*^ control mice with median survivals of 33 days and 41 days, respectively (Fig. 1B-C). Spleen size and weight were increased in moribund *miR196b*^*–/–*^*Dnmt3a*^+/–^*Flt3*^*ITD*^ as compared to age-matched *Dnmt3a*^+/-^*Flt3*^*ITD*^ mice (Fig. 1D, E), indicative of accelerated leukemia development. Flow cytometric analyses comparing leukemic stem/progenitor cells isolated from the bone marrow and spleens of moribund *miR196b*^*–/–*^*Dnmt3a*^+/–^*Flt3*^*ITD*^ and *Dnmt3a*^+/–^*Flt3*^*ITD*^ mice revealed a significant decrease in the total number of lineage negative (Lin^-^) cells in the bone marrow but not the spleen (Fig. 1F, G). No significant differences in the proportion of immunophenotypic common myeloid progenitor (CMP), granulocyte-monocyte progenitor (GMP), or megakaryocyte-erythroid progenitor (MEP) cells were detected in the bone marrow or spleens from moribund-matched *miR196b*^*–/–*^*Dnmt3a*^+/–^*Flt3*^*ITD*^ and *Dnmt3a*^+/–^*Flt3*^*ITD*^ mice (Fig. 1F, G). The proportion of leukemic Lin^-^Sca1^+^c-Kit^+^ (LSK) cells was significantly increased in the bone marrow, but not spleens of moribund *miR196b*^*–/–*^*Dnmt3a*^+/-^*Flt3*^*ITD*^ mice compared to *Dnmt3a*^+/-^*Flt3*^*ITD*^ controls (Fig. 1F, G). These data are consistent with accelerated leukemia development in *miR196b*^*–/–*^*Dnmt3a*^+/–^*Flt3*^*ITD*^ compared to *Dnmt3a*^+/-^*Flt3*^*ITD*^ mice, which was maintained upon transplantation of equal numbers of AML cells into normal healthy secondary recipient mice (Fig. 1H). These data indicate that endogenous miR-196b activity delays *Dnmt3a/Flt3*-mutant leukemia development.

**Fig. 2:**
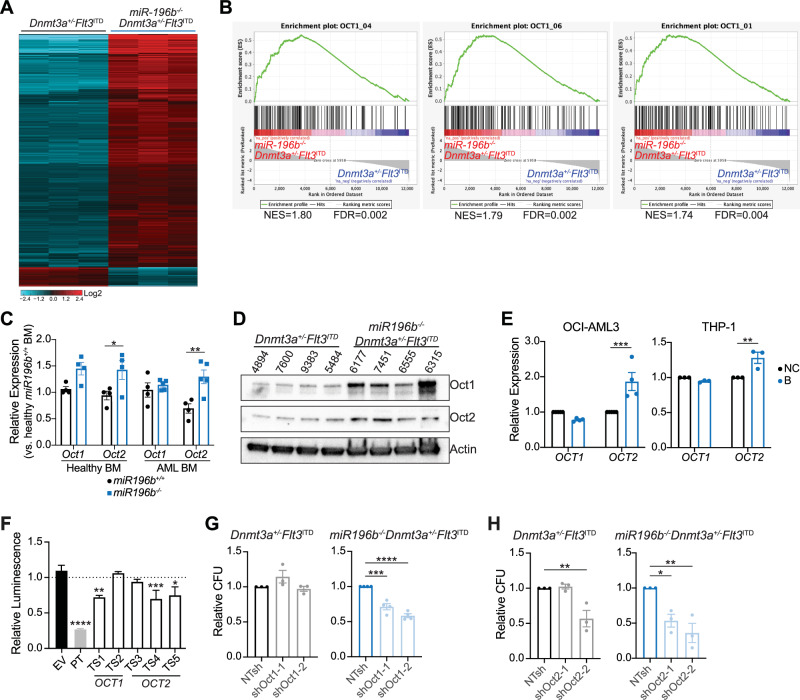
Oct1/2 activity downstream of miR-196b is important for the clonogenicity of *Dnmt3a/Flt3*-mutant AML. **A** Heatmap of differentially expressed genes by RNA-sequencing of c-Kit^+^ AML cells enriched from moribund *miR196b*^*–/–*^*Dnmt3a*^*+/-*^*Flt3*^*ITD*^ (*n* = 3) as compared to *Dnmt3a*^*+/–*^*Flt3*^*ITD*^ (*n* = 3) mice. **B** GSEA enrichment plots of Oct transcription factor target genes enriched in *miR196b*^*–/–*^*Dnmt3a*^*+/–*^*Flt3*^*ITD*^ AML compared *Dnmt3a*^*+/–*^*Flt3*^*ITD*^ AML by RNA-seq. **C** RT-qPCR analysis of average *Oct1* and *Oct2* expression ±SEM in c-Kit^+^ bone marrow *miR-196b*^*–/–*^ (*n* = 4) compared to wild-type (*n* = 4) and *miR196b*^*–/–*^*Dnmt3a*^*+/–*^*Flt3*^*ITD*^ AML (*n* = 4) compared to *Dnmt3a*^*+/–*^*Flt3*^*ITD*^ AML (*n* = 5). Significance determined by two-way ANOVA Šídák’s multiple comparisons test. **D** Representative Western blot of individual *Dnmt3a*^*+/–*^*Flt3*^*ITD*^ (*n* = 4) and *miR196b*^*–/–*^*Dnmt3a*^*+/–*^*Flt3*^*ITD*^ (*n* = 4) AML for Oct1 and Oct2. Actin serves as loading control. **E** RT-qPCR analysis of relative *OCT1* and *OCT2* expression ± SEM of anti-miR-196b morpholino treated OCI-AML3 (*n* = 3) and THP-1 (*n* = 3) human cell lines. Significance was determined by two-way ANOVA Šídák’s multiple comparisons test. **F** Average luminescence relative to EV control ±SEM from dual luciferase reporter assays conducted in HEK293T cells of miR-196b target sites from *OCT1* and *OCT2* (*n* = 3–6 experimental replicates). Significance was evaluated by one-way ANOVA Dunnett’s multiple comparisons test. **G** Average number of colonies (CFU) ± SEM formed by *Dnmt3a*^*+/–*^*Flt3*^*ITD*^ (*n* = 3) or *miR196b*^*-/-*^*Dnmt3a*^*+/–*^*Flt3*^*ITD*^ AML cells (*n* = 4) expressing shOct1-1 or shOct1-2 relative to each respective experimental NTsh control. Significance evaluated by one-way ANOVA Šídák’s multiple comparisons test. **H** Average number of colonies (CFU) ± SEM formed by *Dnmt3a*^*+/–*^*Flt3*^*ITD*^ (*n* = 3) or *miR196b*^*–/–*^*Dnmt3a*^*+/–*^*Flt3*^*ITD*^ AML cells (*n* = 3) expressing shOct2-1 or shOct2-2, relative to each respective experimental NTsh control. Significance evaluated by one-way ANOVA Šídák’s multiple comparisons test. For all panels, ****p < 0.0001, ***p < 0.001, **p < 0.01, *p < 0.05.

To understand the mechanism(s) of accelerated leukemogenesis in *miR196b*^*–/–*^*Dnmt3a*^+/–^*Flt3*^*ITD*^ mice, we performed RNA-seq on c-Kit^+^ AML cells isolated from moribund *miR196b*^*-/-*^*Dnmt3a*^+/-^*Flt3*^*ITD*^ and *Dnmt3a*^+/-^*Flt3*^*ITD*^ mice as controls. We identified 3119 significantly differentially expressed genes, of which 2,882 were upregulated in *miR196b*^*-/-*^*Dnmt3a*^+/-^*Flt3*^*ITD*^ AML (Fig. 2A and Supplementary Table 1). Gene set enrichment analysis (GSEA) identified significant enrichment for target genes of HoxA9/Meis1, suggesting deregulation of known miR-196b target HoxA9 despite no significant changes in either *HoxA9* or *Meis1* expression (Supplementary Fig. 2A and Supplementary Table 1). Target genes of the POU domain-containing family class II of transcription factors are also upregulated in *miR196b*^*–/–*^*Dnmt3a*^+/–^*Flt3*^*ITD*^ AML cells (Fig. 2B and Supplementary Table 1). We detect significant upregulation of Oct1 and Oct2, members of the POU II class, at the protein level and *Oct2* mRNA, in *miR196b*^*–/–*^*Dnmt3a*^+/–^*Flt3*^*ITD*^ as compared to *Dnmt3a*^+/–^*Flt3*^*ITD*^ AML cells (Fig. 2C, D and Supplementary Fig. 2B) and *miR-196b*^*–/–*^ versus wild-type bone marrow (Fig. 2C). Treatment of human AML cells with morpholinos directed against miR-196b significantly elevated *OCT2*, but not *OCT1*, mRNA levels (Fig. 2E and Supplementary Fig. 2C). Intersection of genes upregulated in *miR196b*^*–/–*^*Dnmt3a*^+/–^*Flt3*^*ITD*^ AML by RNA-seq with miR-196b predicted targets and previously published experimentally defined miR-196b targets in MLL-rearranged AML [9] further support Oct1 as a potential target of miR-196b (Supplementary Fig. 2D). Given that miRNA can mediate mRNA target decay and/or inhibit translation, these data are consistent with the possibility that miR-196b regulates OCT1 and OCT2. Next, we measured the ability miR-196b to repress the expression of a luciferase reporter fused to putative target sites in *OCT1* and *OCT2* (Supplementary Fig. 2E). In the presence of miR-196b mimics, one *OCT1* and two *OCT2* target site reporters were significantly repressed by miR-196b (Fig. 2F), suggesting that miR-196b can bind to putative target site sequences in *OCT1* and *OCT2*. Oct1, also known as Pou2f1, and Oct2, also known as Pou2f2, are established co-regulators of B-cell development and gene expression [10], but more recently roles have emerged in myeloid biology and malignancies. It was recently reported that Oct1 may protect hematopoietic stem and progenitor cells (HSPCs) from proliferative stress and was required for AML development mediated by the fusion oncoprotein MLL-AF9 [9, 11]. Oct2 was recently found to co-regulate transcriptional programs induced upon TLR8-mediated neutrophil activation [12]. To test if Oct1 or Oct2 are important for *Dnmt3a/Flt3*-mutant AML self-renewal and/or proliferation and if *miR196b*^*–/–*^*Dnmt3a*^+/–^*Flt3*^*ITD*^ AML is preferentially sensitive to the levels of these transcription factors, we expressed two independent shRNA against *Oct1* and *Oct2* in *Dnmt3a*^+/–^*Flt3*^*ITD*^ and *miR196b*^*–/–*^*Dnmt3a*^+/–^*Flt3*^*ITD*^ AML. Relative to non-targeting (NTsh) control shRNA, *Oct1* and *Oct2* knockdown significantly reduced the colony forming potential of *miR196b*^*–/–*^*Dnmt3a*^+/–^*Flt3*^*ITD*^ AML, but not *Dnmt3a*^+/–^*Flt3*^*ITD*^ AML (Fig. 2G, H and Supplementary Fig. 2F, G), suggesting that the deletion of miR-196b creates a unique requirement for Oct1 and Oct2 in *miR196b*^*-/-*^*Dnmt3a*^+/-^*Flt3*^*ITD*^ AML. These data are consistent with the notion that Oct1 and Oct2 support the more aggressive *miR196b*^*–/–*^*Dnmt3a*^+/–^*Flt3*^*ITD*^ AML phenotype.

We and others have shown that miR-196b is important for the proliferation, survival, and differentiation block in *MLL*-rearranged and other *HOXA*-gene signature AML [9, 13, 14]. *DNMT3A* loss-of-function mutations are common in AML, and result in enhanced HSPC self-renewal, and cause DNA hypomethylation and overexpression of miR-196b [3, 7, 15]. We establish herein a novel protective role for miR-196b in HSPCs that delays AML development driven by coincident *Dnmt3a* and *Flt3*^*ITD*^ mutations whereby *Dnmt3a/Flt3*-mutant mice with deletion of miR-196b succumb to AML faster than *Dnmt3a/Flt3*-mutant mice with endogenous miR-196b. Although this contrasts with our previous report describing a pro-leukemic role for miR-196b in already established *DNMT3A/FLT3*-mutant AML [7], a similar dichotomy was revealed for miR-196b in MLL-r AML [13]. Taken together, there are differing requirements for miR-196b expression at different stages of AML development and miR-196b targets genes with both pro- and anti-leukemic roles. We cannot discount the possibility that loss of miR-196b from non-hematopoietic cells could have a role in accelerated AML development since our mice carry miR-196b deletion in the germline. It is also possible that miR-196b activity may alter *Flt3*^ITD^ signaling to influence disease pathogenesis. Future studies will need to address these possibilities. Previously, we identified that miR-196b blocks differentiation and maintains the immature state of human and murine *DNMT3A/FLT3*-mutant AML blasts in response to pathogenic stimuli through repression of TLR7/8 signaling [7]. In this study, we identify POU domain-containing class II transcription factor signatures, namely Oct1 and Oct2, associated with the more aggressive *miR196b*^*-/-*^*Dnmt3a*^+/-^*Flt3*^*ITD*^ AML phenotype. Given the emerging evidence for Oct1 in HSPCs and leukemic transformation and Oct2 in activating TLR-mediated inflammatory signaling in myeloid cells, this putative miR-196b/Oct1/2 signaling axis could be an important ‘switch’ in controlling HSPC cell fate upon acquisition of AML driver mutations such as *DNMT3A*. Altogether, our data indicate miR-196b is a pivotal and pleiotropic regulator of myeloid leukemogenesis.

## Supplementary information

Supplementary Methods and Figure Legends

Supplementary Figure 1

Supplementary Figure 2

Supplementary Table
