# Supplementary material for: miR-196b-Oct1/2 axis regulates DNMT3A-mutant AML pathogenesis
Source: Leukemia. 2024 Nov 23;39(1):229–33. doi: 10.1038/s41375-024-02456-8 (PMC11717699; doi:10.1038/s41375-024-02456-8)
Supplement: Supplementary file 1 — Supplementary Methods and Figure Legends [file 41375_2024_2456_MOESM1_ESM.pdf]

Supplementary Information

Methods

Mice

The generation of *miR-196b*$^{-/-}$ mice was described previously(1) and are maintained on a C57BL6/J background. For normal steady state hematopoiesis studies wild-type (WT), *miR196b*$^{+/-}$, and miR-196b$^{-/-}$ littermates were used between 7-10 weeks of age. To study AML, *Flt3*$^{ITD/ITD}$ mice(2) were bred with *Dnmt3a*$^{fl/fl}$ mice(3) with and without *Mx1-Cre*(4) to generate *Flt3*$^{ITD/ITD}$*;Dnmt3a*$^{fl/fl}$ *Mx1-Cre* (referred to as *Dnmt3a*$^{+/-}$*Flt3*^{ITD}) mice and *Flt3*$^{ITD/ITD}$*;Dnmt3a*$^{fl/fl}$ (referred to as *Flt3*^{ITD}) mice as reported previously(5). As previously published, *Dnmt3a*$^{+/-}$*Flt3*^{ITD} mice are not treated with pIpC, but instead hematopoietic cells are allowed to spontaneously delete one or both alleles of *Dnmt3a* resulting in lethal AML, while *Flt3*^{ITD} mice with wild-type *Dnmt3a* develop a non-lethal myeloproliferative neoplasm (MPN)(5). *Flt3*$^{ITD/ITD}$*;Dnmt3a*$^{fl/fl}$*;miR196b*$^{-/-}$ *Mx1-Cre* (referred to as *miR196b*$^{-/-}$ *Dnmt3a*$^{+/-}$*Flt3*^{ITD} AML) mice were generated by crossing *Flt3*$^{ITD/ITD}$*;Dnmt3a*$^{fl/fl}$ *Mx1-Cre* mice with *miR-196b*$^{-/-}$ mice. *miR196b*$^{-/-}$*Dnmt3a*$^{+/-}$*Flt3*^{ITD} AML mice are also not treated with pIpC are allowed to spontaneously delete one or both *Dnmt3a* alleles resulting in rapidly lethal AML. All mice are maintained on a CD45.2^{+} C57BL6/J background. Spleens were isolated from moribund *miR196b*$^{-/-}$*Dnmt3a*$^{+/-}$*Flt3*^{ITD} AML and age-matched *Dnmt3a*$^{+/-}$ *Flt3*^{ITD} AML mice for comparison of size and weight. For secondary transplants, CD45.1^{+} C57BL6/J mice (Charles River) were irradiated with 700 Rads prior to tail-vein injection of 1 million c-Kit^{+} AML cells isolated from bones of moribund *miR196b*$^{-/-}$*Dnmt3a*$^{+/-}$*Flt3*^{ITD} and *Dnmt3a*$^{+/-}$*Flt3*^{ITD} mice. All mice above were housed, bred, and utilized at Thomas

Jefferson University according to approved Institutional Animal Care and Use Committee protocols (IACUC protocol #01932).

Pathology

Sternum, spleen, and liver were isolated from moribund *miR-196b*$^{-/-}$*Dnmt3a*$^{+/-}$*Flt3*^{ITD}, age-matched *Dnmt3a*$^{+/-}$*Flt3*^{ITD}, and WT mice. The sternums were decalcified, and all organ samples were fixed with paraffin and stained with hematoxylin & eosin (H&E) by the SKCC Translational Research/Pathology Core. Images were taken on Aperio Scanscope CS2 microscope and analyzed using ImageScope v12.4.6.5003.

Flow Cytometric Analyses and Cell Enrichments

Equal numbers of bone marrow, spleen, or peripheral cells were washed in 1X FACS buffer (1% FBS and 0.01% sodium azide in DPBS) after red blood cell lysis and stained with the fluorochrome conjugated antibodies as previously described(5). All flow cytometric analyses were performed on FACS LSR Fortessa or Symphony A5 (BD Biosciences). Data analyses were performed using FlowJo software.

All antibodies were purchased from BioLegend unless otherwise stated. Live cells were distinguished using DAPI viability stain. For normal and leukemic stem/progenitor cell analyses, gated as previously described(5), mouse bone marrow cells and leukemic splenocytes were first stained with biotin-conjugated antibodies against CD3 (clone 145-2C11), CD4 (clone RM4-5), CD8 (53-6.7), CD11b (clone M1/70), Gr1 (clone RB6-8C5), Ter119 (clone TER-119), CD45R (clone RA3-6B2), CD19 (clone 6D5), CD127 (clone A7R43), and PerCP-Cy5.5-conjugated anti-CD16/32 (clone 93). PE-Cy7-conjugated anti-Sca1 (clone D7), APC-conjugated anti-CD117 (clone 2B8), and BV421-conjugated anti-CD34 (clone RAM34) antibodies were also used in this first stain. Cells were then washed

and stained with streptavidin-conjugated APC-Cy7. For mature myeloid and lymphoid peripheral blood analyses, red blood cells were first lysed. Cells were then washed and stained with AF-700-conjugated anti-B220 (clone RA3-6B2), BV421-conjugated anti-CD11b (clone M1/70), APC-conjugated anti-CD3 (clone 500A2), and PE-Cy7-conjugated anti-Gr1 (clone RB6-8C5) antibodies.

c-Kit^{+} enrichments of bone marrow or spleen AML cells were performed using CD117 microbeads according to manufacturer's instructions (Miltenyi BioTec) using AutoMACS Pro separator or magnetic stand (Miltenyi BioTec).

Total RNA Sequencing Analysis

RNA was purified by TriZol from c-Kit^{+} AML splenocytes harvested from moribund *miR196b*$^{-/-}$*Dnmt3a*$^{+/-}$*Flt3*^{ITD} mice. cDNA libraries were sequenced at 30 million reads per sample. Data were processed together with previously generated RNA-seq on c-Kit^{+} AML splenocytes harvested from moribund *Dnmt3a*$^{+/-}$*Flt3*^{ITD} mice(5) (GSE77846) and differential gene expression analyses performed using AltAnalyze 2.1.3(6). The "DATASET" expression output file was used to generate a pre-ranked gene list wherein all expressed genes were ranked by -log10(p-value)*sign(fold-change) then analyzed using GSEAPreranked v4.3.3(7).

Cell Culture

HEK293T cells were cultured at 100,000 cells/mL in DMEM (Life Technologies) with 10% heat inactivated FBS and 1% penicillin and streptomycin (Life Technologies). OCI-AML3 and THP-1 cell lines (DSMZ) were cultured at 500,000 cells/mL in alpha-MEM plus nucleosides (Life Technologies) or RPMI (Life Technologies) with 10% heat-inactivated FBS and 1% penicillin and streptomycin. OCI-AML3 and THP-1 cell lines were plated at

a concentration of 500,000 cells/mL and incubated for 72 hours with 8µM and 4µM of anti-miR-196b morpholino, respectively, and standard negative control vivo morpholino.

Vivo Morpholino Sequences

miR-196b targeting vivo morpholino and the vivo morpholino standard negative control were purchased from GeneTools.

Anti-miR-196b (B): 5'- ATCCCAACAACAGGAAACTACCTAA –3'

Dual Luciferase Reporter Assay

miR-196b target binding sites (TS) were predicted within the mRNA transcripts of *OCT1* isoform 1 (NM_002697.4) and *OCT2* isoform 1 (NM_001207025.4), cloned into the psiCHECK2 dual luciferase reporter vector (Promega), and sequenced for correct inserts. Dual luciferase reporter assays were performed as described previously(8). The following oligos were used for cloning into psiCHECK2:

OCT1-TS1:

5'-

GCCCCAGGGCCAGCAGGGTCTCCTGCAAGCGCAAAATCTTCTAACGCAACTACCT

CAGCAAAGCCAAGCCAACCTCCTACAGTCGCAGCC –3'

OCT1-TS2:

5'-

ACCAACATCCGTGTGGCCTTAGAGAAGAGTTTCTTGGAGAATCAAAAGCCTACCTC

GGAAGAGATCACTATGATTGCTGATCAGCTCAAT –3'

OCT2-TS3:

5'-

GGCCCAGCAGAGCCAGCCAGGCCTGCTACCGACACCAAATCTATTCCAGCTACCT

CAGCAAACCCAGGGAGCTCTTCTGACCTCCCAGCC –3'

OCT2-TS4:

5’-

ACAAACGTCCGCTTCGCCTTAGAGAAGAGTTTTCTAGCGAACCAGAAGCCTACCTC

AGAGGAGATCCTGCTGATCGCCGAGCAGCTGCAC –3'

OCT2-TS5:

5’-

GGGACCTTACCGTTGTCCCAAGCTTCCAGCAGTCTGAGCACAACAGTTACTACCTT

ATCCTCAGCTGTGGGGACGCTCCACCCCAGCCGG –3'

shRNA Cloning

pLKO.1-puro (#8453 Addgene) was digested with *Kpn*1 and *Bam*HI to replace the puromycin cassette with mVenus to generate pLKO.1-mVenus. Then, *Age*I and *Eco*RI were used to remove the 1.9kb stuffer sequence and replaced with shOct1-1 (TRCN0000240636), shOct1-2 (TRCN0000240640), shOct2-1 (TRCN0000245326), or shOct2-2 (TRCN0000020820). The non-targeting shRNA control plasmid (SHC002 Sigma) was digested with *Kpn*1 and *Bam*HI to replace the puromycin cassette with mVenus.

shRNA Lentivirus Production and Transduction

For virus production, Lenti-X 293T (Clonetech) were seeded at $14x10^6$/T175 flask in DMEM (ThermoFisher Scientific) with 10% heat-inactivated FBS (Atlanta Biologics) and L-Glutamine (ThermoFisher Scientific). The following day, 50 µg of lentiviral plasmid DNA was cotransfected with 50 µg of psPAX2 and 10 µg of VsVG (#14888. Addgene) into Lenti-X 293T cells using Transit-LT1 (Mirus) according to manufacturer’s protocol. 6-7

hours post transfection, the transfection media was carefully discarded and replaced with DMEM (ThermoFisher Scientific) with 30% heat-inactivated FBS (Atlanta Biologics), L-Glutamine (ThermoFisher Scientific) and penicillin-streptomycin (ThermoFisher Scientific). Virus containing supernatant was collected 48 and 72 hours after transfection, pooled, and concentrated using Lenti-X Concentrator (Takara) according to manufacturer's instructions and frozen at -80ºC in aliquots.

Prior to transduction, culture dishes were treated with 50μg/ml Retronectin (Takara) overnight at 4 ºC, then washed prior to addition and spinning of virus for 30 minutes at 1,500RCF at 30°C. c-Kit^{+} *miR196b*$^{-/-}$*Dnmt3a*$^{+/-}$*Flt3*^{ITD} and *Dnmt3a*$^{+/-}$*Flt3*^{ITD} AML cells suspended in culture media with recombinant mouse SCF (60ng/ml), IL-6 (20ng/ml), and IL-3 (20ng/ml) (Miltenyi BioTec) and hexadimethrine bromide/polybrene (Sigma) were added and spun with virus for 90 minutes at 1,500RCF at 30°C then resuspended in culture media and incubated for 48 hours. Cells were isolated from culture and washed prior to FACS sorting for mVenus+ transduced cells, which were collected for CFU assay or for RT-qPCR analysis.

Colony Forming Assays

For shRNA CFU, 3,000 transduced c-Kit^{+} *miR196b*$^{-/-}$*Dnmt3a*$^{+/-}$*Flt3*^{ITD} and *Dnmt3a*$^{+/-}$*Flt3*^{ITD} AML cells were plated in triplicate in Methocult GF M3434 (Stemcell Technologies). After 6 days, total colonies were enumerated.

RNA Isolation, cDNA Synthesis, and RT-qPCR

RNA was isolated by TriZol extraction (Life Technologies) or by RNeasy Micro kit (Qiagen) according to manufacturer's protocol. cDNA was synthesized using the High-Capacity cDNA kit (Life Technologies) with random primers for standard gene expression

analysis or miRNA-specific primers for miRNA expression analysis. Gene and miRNA expression were quantified on QuantStudio 3 by TaqMan (ThermoFisher) qPCR using the delta-delta Ct method for *hsa-miR-196b-5p* (#002215), *hsa-miR-196a-5p* (#241070), *Oct1/Pou2f1* (Mm00448332_m1), *Oct2/Pou2f2* (Mm00448354_m1), *OCT1/POU2F1* (Hs01552829_m1), and *OCT2/POU2F2* (Hs00922172_m1). Mouse U6 snRNA (#001973), *Sdha* (Mm01352366_m1), and human RNU6B (#001093) and *SDHA* (Hs00417200_m1) served as the housekeeping controls.

Western Blot Analysis

Equal number of spleen cells from four individual *Dnmt3a*$^{+/-}$*Flt3*^{ITD} mice and four individual *miR196b*$^{-/-}$*Dnmt3a*$^{+/-}$*Flt3*^{ITD} mice were washed in PBS and lysed in RIPA buffer plus protease inhibitor. Equal amounts of protein lysates were separated on Bis-Tris 4-12% gradient gels (NuPage) and transferred to PVDF membranes using an iBlot2 (Invitrogen). Membranes were blocked in 5% milk in 1x TBST for 45 minutes and then incubated with primary antibodies to detect: OCT1 (clone JG62-35, Invitrogen), OCT2 (clone 18996-I-AP, Proteintech), and β-actin (AC-15, Sigma). Membranes were washed, then incubated with HRP conjugated anti-rabbit or anti-mouse antibodies, then washed, and incubated with ECL and exposed using a BioRad ChemiDoc MP Imaging System. Relative band intensity was quantified using ImageJ, with each Oct protein normalized to the respective β-actin loading control.

Data Sharing Statement

RNA-sequencing data of murine *Dnmt3a*$^{+/-}$*Flt3*^{ITD} and *miR196b*$^{-/-}$*Dnmt3a*$^{+/-}$*Flt3*^{ITD} can be accessed in NCBI's Gene Expression Omnibus (GSE77846 and GSE192639). Data were analyzed as previously described(5, 9).

Statistics

Statistical analyses were performed using Prism 9.2.0. Statistical tests performed, as appropriate, include log-rank (Mantel-Cox) test of Kaplan-Meier survival curves, unpaired t-tests, ordinary one-way ANOVA, or two-way ANOVA with multiple comparisons tests as needed. See figure legends for details.

Supplementary Figure Legends

Supplementary Figure 1. Loss of miR-196b does not alter normal hematopoiesis. A) Total bone marrow count ±SEM of WT, *miR196b*$^{+/-}$, and *miR196b*$^{-/-}$ mice. **B-F)** Flow cytometric analyses of **B)** Lin-, **C)** LSK, **D)** CMP, **E)** GMP, and **F)** MEP populations in the bone marrow of WT, *miR196b*$^{+/-}$, and *miR196b*$^{-/-}$ mice illustrated as a percent of total bone marrow cells ±SEM. **G-H)** Flow cytometric analyses of the proportions of **G)** myeloid and **H)** lymphoid cell types in peripheral blood ±SEM of WT, *miR196b*$^{+/-}$, and *miR196b*$^{-/-}$ mice. Significant differences were evaluated by ordinary one-way ANOVA.

Supplementary Figure 2. Loss of miR-196b deregulates targets involved in leukemogenesis. A) GSEA enrichment plot of MEIS1A/HOXA9 transcription factor target genes enriched in *miR196b*$^{-/-}$*Dnmt3a*$^{+/-}$*Flt3*^{ITD} AML compared *Dnmt3a*$^{+/-}$*Flt3*^{ITD} AML by RNA-seq. **B)** Average band intensities ±SEM of Oct1 and Oct2 relative to β-actin as the loading control in *Dnmt3a*$^{+/-}$*Flt3*^{ITD} (n=4) and *miR196b*$^{-/-}$*Dnmt3a*$^{+/-}$*Flt3*^{ITD} (n=4) AML. Significant differences determined by unpaired t-tests with multiple corrections at 1% FDR (two-stage step-up Benjamini, Krieger, and Yekutieli). **C)** RT-qPCR analysis of relative miR-196b expression ±SEM of OCI-AML3 (n=3) and THP-1 (n=3) human cell lines treated

with anti-miR196b morpholino. Experiment was performed in triplicate. Significance determined by unpaired t-test. **D)** Venn diagram intersection of genes upregulated in *miR196b*$^{-/-}$*Dnmt3a*$^{+/-}$*Flt3*^{ITD} AML, predicted miR-196b targets, and miR-196b pulldown targets either enriched or depleted in *in vivo* MLL-AF9 AML shRNA screen (see Methods). **E)** Sequence alignment of miR-196b with putative target sites (TS) in human and murine *POU2F1* and *POU2F2*. **F-G)** Average *Oct1* **(F)** and *Oct2* **(G)** expression ±SEM by RT-qPCR for each shRNA condition relative to each respective experimental NTsh control, represented by the dotted line indicating a relative expression of 1, in *miR196b*$^{-/-}$*Dnmt3a*$^{+/-}$*Flt3*^{ITD} AML (n=4) or *Dnmt3a*$^{+/-}$*Flt3*^{ITD} AML (n=3) cells. Significance determined by one-way ANOVA Šídák's multiple comparisons test.

References

1. Wong SF, Agarwal V, Mansfield JH, Denans N, Schwartz MG, Prosser HM, et al. Independent regulation of vertebral number and vertebral identity by microRNA-196 paralogs. Proc Natl Acad Sci U S A. 2015;112(35):E4884-93.
2. Lee BH, Tothova Z, Levine RL, Anderson K, Buza-Vidas N, Cullen DE, et al. FLT3 mutations confer enhanced proliferation and survival properties to multipotent progenitors in a murine model of chronic myelomonocytic leukemia. Cancer cell. 2007;12(4):367-80.
3. Kaneda M, Okano M, Hata K, Sado T, Tsujimoto N, Li E, et al. Essential role for de novo DNA methyltransferase Dnmt3a in paternal and maternal imprinting. Nature. 2004;429(6994):900-3.
4. Kuhn R, Schwenk F, Aguet M, Rajewsky K. Inducible gene targeting in mice. Science. 1995;269(5229):1427-9.
5. Meyer SE, Qin T, Muench DE, Masuda K, Venkatasubramanian M, Orr E, et al. DNMT3A Haploinsufficiency Transforms FLT3ITD Myeloproliferative Disease into a Rapid, Spontaneous, and Fully Penetrant Acute Myeloid Leukemia. Cancer Discov. 2016;6(5):501-15.
6. Emig D, Salomonis N, Baumbach J, Lengauer T, Conklin BR, Albrecht M. AltAnalyze and DomainGraph: analyzing and visualizing exon expression data. Nucleic Acids Res. 2010;38(Web Server issue):W755-62.
7. Subramanian A, Tamayo P, Mootha VK, Mukherjee S, Ebert BL, Gillette MA, et al. Gene set enrichment analysis: a knowledge-based approach for interpreting genome-wide expression profiles. Proc Natl Acad Sci U S A. 2005;102(43):15545-50.

8. Gamlen HA, Romer-Seibert JS, Lawler ME, Versace AM, Goetz ML, Feng Y, et al. miR-196b-TLR7/8 Signaling Axis Regulates Innate Immune Signaling and Myeloid Maturation in DNMT3A-Mutant AML. Clin Cancer Res. 2022;28(20):4574-86.
9. Meyer SE, Muench DE, Rogers AM, Newkold TJ, Orr E, O'Brien E, et al. miR-196b target screen reveals mechanisms maintaining leukemia stemness with therapeutic potential. J Exp Med. 2018;215(8):2115-36.
