# Supplementary figures and images for: miR-196b-Oct1/2 axis regulates DNMT3A-mutant AML pathogenesis
Source: Leukemia. 2024 Nov 23;39(1):229–33. doi: 10.1038/s41375-024-02456-8 (PMC11717699; doi:10.1038/s41375-024-02456-8)

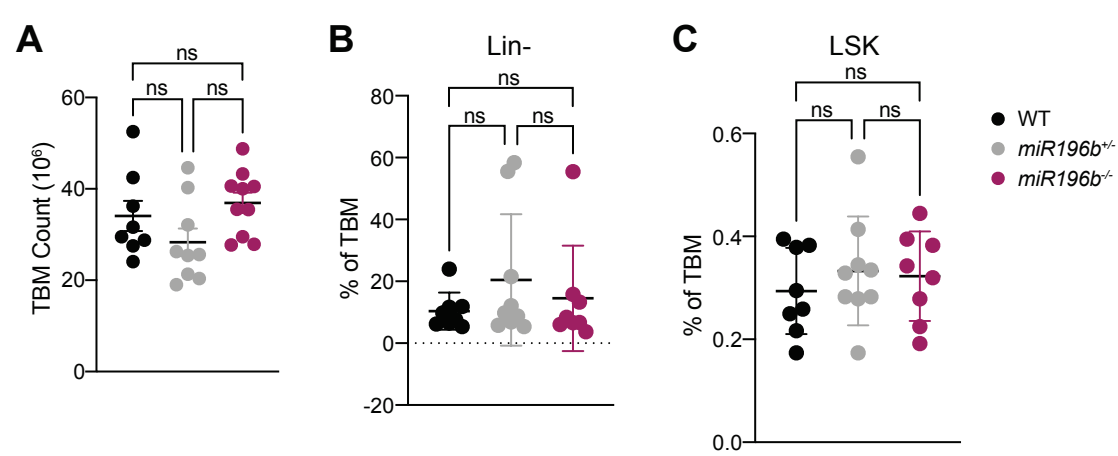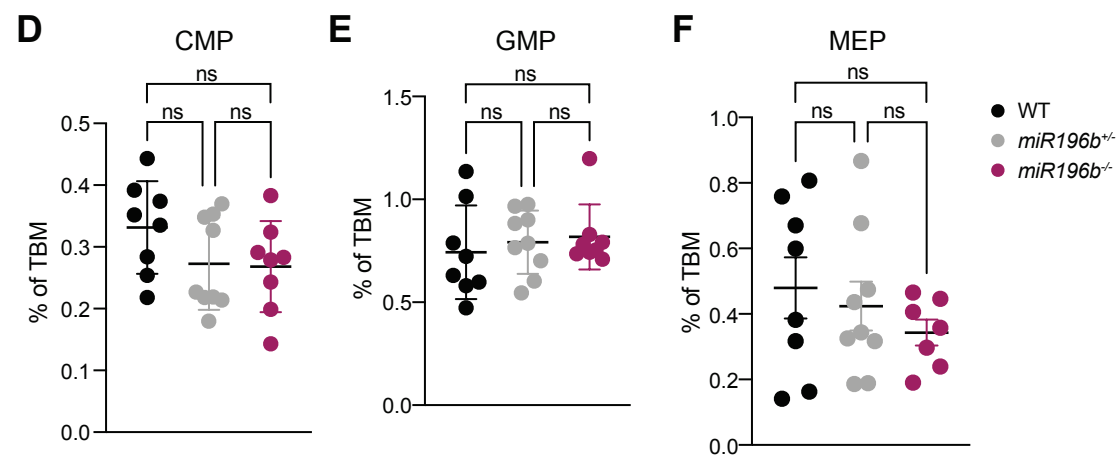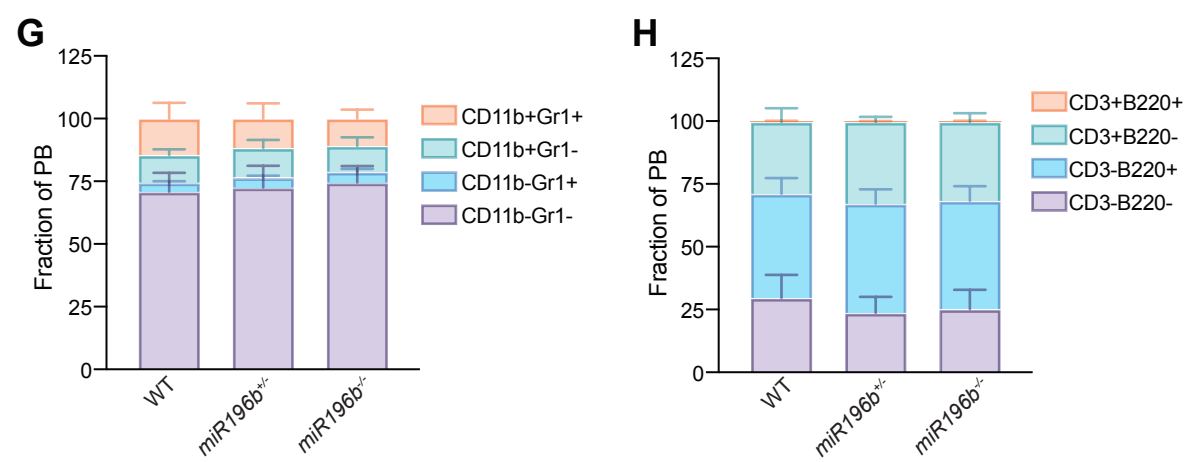

Supplement: Supplementary file 2 — Supplementary Figure 1 [file 41375_2024_2456_MOESM2_ESM.pdf]

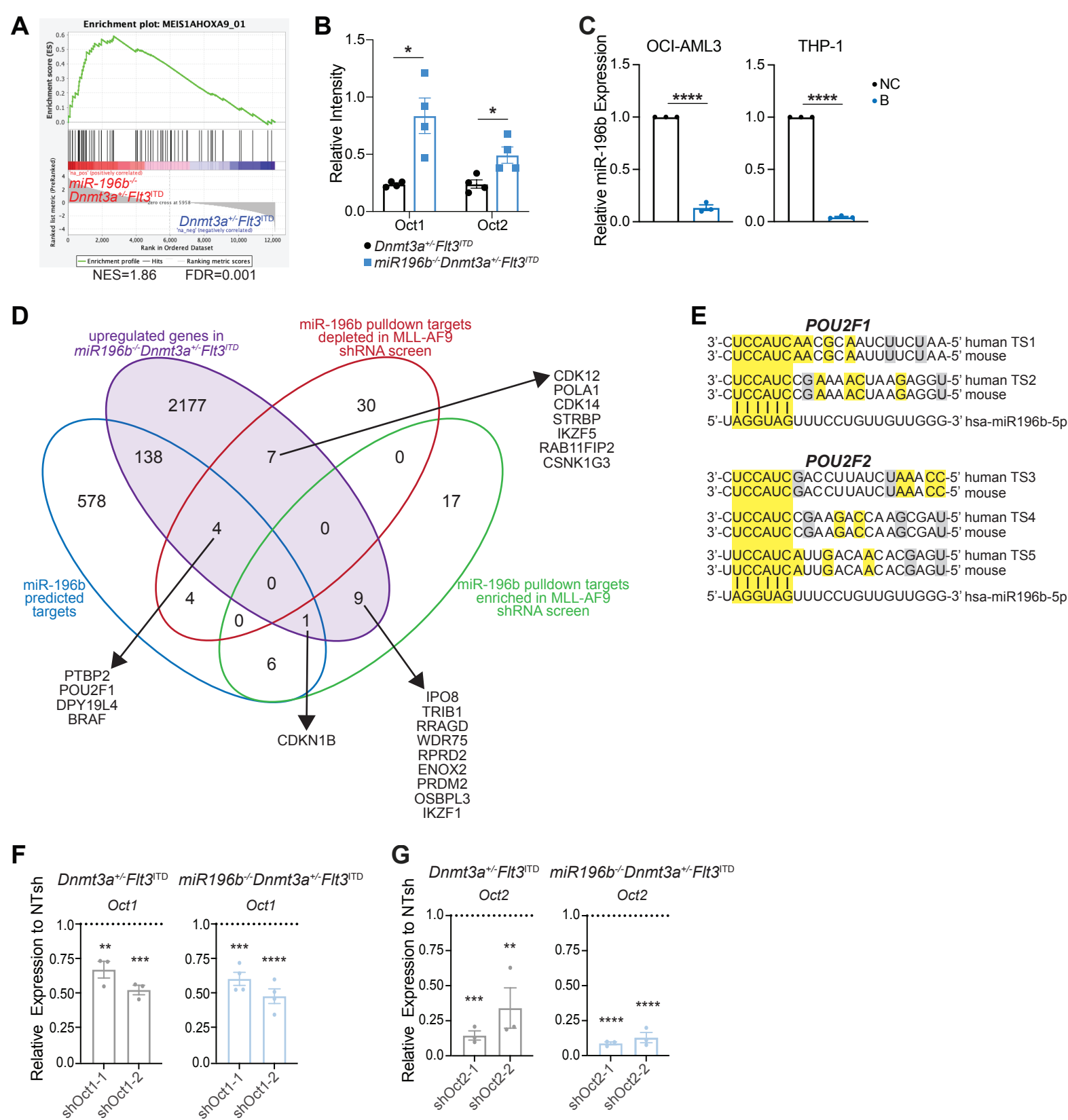

Supplement: Supplementary file 3 — Supplementary Figure 2 [file 41375_2024_2456_MOESM3_ESM.pdf]
